# Novel Loss-of-Function Variants in *CHD2* Cause Childhood-Onset Epileptic Encephalopathy in Chinese Patients

**DOI:** 10.3390/genes13050908

**Published:** 2022-05-19

**Authors:** Xu Wang, Di Cui, Changhong Ding, Chunhong Chen, Xiaohui Wang, Fang Fang, Hong Jin, Xiaotun Ren

**Affiliations:** 1 Department of Neurology, Beijing Children’s Hospital, Capital Medical University, National Centre for Children’s Health, Beijing 100045, China; 13641290689@163.com (C.D.); sjkbch@sina.com (C.C.); heqi50@sina.com (X.W.); 13910150389@163.com (F.F.); jinhongjh@126.com (H.J.); rxt1997@126.com (X.R.); 2 Running Gene Inc., Beijing, 100085, China; cuidibiology@163.com

**Keywords:** epileptic encephalopathy (EE), chromodomain-helicase-DNA-binding protein 2 (CHD2), childhood-onset epileptic encephalopathy (DEE94), seizure, developmental delay, intellectual disability (ID)

## Abstract

Developmental and epileptic encephalopathy-94 (DEE94) is a severe form of epilepsy characterized by a broad spectrum of neurodevelopmental disorders. It is caused by pathogenic *CHD2* variants. While only a few pathogenic *CHD2* variants have been reported with detailed clinical phenotypes, most of which lack molecular analysis. In this study, next-generation sequencing (NGS) was performed to identify likely pathogenic *CHD2* variants in patients with epilepsy. Three likely pathogenic variants were finally identified in different patients. The seizure onset ages were from two years to six years. Patients 1 and 2 had developmental delays before epilepsy, while patient 3 had intellectual regression after the first seizure onset. The observed seizures were myoclonic, febrile, and generalized tonic-clonic, which had been controlled by different combinations of antiepileptic drugs. Two *de novo* (c.1809_1809+1delGGinsTT, p.? and c.3455+2_3455+3insTG, p.?) and one maternal (c.3783G>A, p.W1261*) variant were identified, which were all predicted to be pathogenic/likely pathogenic. Molecular analysis was performed in patient 1, and we detected aberrantly spliced products, proving the pathogenicity of this *CHD2* variant. New cases with novel variants, along with a detailed clinical and molecular analysis, are important for a better understanding of *CHD2*-related epileptic encephalopathy.

## 1. Introduction

The term “epileptic encephalopathy” (EE) was first proposed in the 1970s to describe devastating epilepsies in early life, which was formally recognized in 2001 by the International League Against Epilepsy (ILAE) [1]. The EEs often occur at a critical period of brain development. Epileptiform activity might contribute to behavioral, cognitive, and or motor slowing or regression [2]. With the application of next-generation sequencing (NGS), variants of several genes have been reported to be responsible for EEs [3], and the gene *CHD2* (MIM 302119; NM_001271.3) on chromosome 15q26 is included [4]. *CHD2* encodes the chromodomain-helicase-DNA-binding protein 2, which belongs to the chromodomain helicase DNA-binding family and is important for regulating gene expression by remodeling chromatin structure and influencing histone acetylation [5]. Although the functional studies of CHD2 are limited, it has been reported that pathogenic variants of *CHD2* would lead to developmental and epileptic encephalopathy-94 (DEE94, OMIM 615369) [6]. DEE94 is a severe form of epilepsy, with a predicated incidence of 6.84 cases per 100,000 live births [7]. It is characterized by a broad spectrum of neurodevelopmental disorders, such as epilepsy, global developmental delay, intellectual disability (ID), and autism spectrum disorder (ASD) [8]. Other clinical phenotypes include photosensitivity and psychomotor regression. The phenotypes caused by *CHD2* variants can be highly variable except epilepsy. Most patients have multiple seizure types and an abnormal electroencephalogram (EEG). Seizures of some patients could be well controlled, while others are refractory to treatment, even with multiple anti-epileptic drugs. To date, there is no data for an exact effective drug for patients with *CHD2* variants. In 2009, *CHD2* variants were reported to cause epilepsy [3]. To date, 86 disease-causing mutations have been reported (according to the Human Mutation Database, HGMD). However, only a few patients with *CHD2* variants have been reported with detailed clinical phenotypes, most of which are *de novo*. The description of the full phenotypic spectrum is lacking and the study of interfamilial phenotypic heterogeneity and penetrance could be difficult.

In this report, we performed genetic analyses of the *CHD2* gene in patients with epilepsy to identify new variants involved and to investigate the phenotypic spectrum associated with variants.

## 2. Materials and Methods

### 2.1. Participants

Three patients with epilepsy who were admitted to the Beijing Children’s Hospital of Capital Medical University from May 2016 to September 2021 and underwent genetic analysis were found to have likely pathogenic *CHD2* variants that were not previously described in the literature. In this report, we describe the clinical presentation and molecular features of these three patients. This study was approved by the Ethics Committee of Beijing Children’s Hospital, and written informed consent was obtained from the parents for genetic analysis and publication of the patients’ clinical and genetic information.

### 2.2. Genetic Analysis

The exome-targeted next-generation sequencing (NGS) of the patients was performed as previously described [9]. DNA was isolated and fragmented by sonication and processed using KAPA HTP Library Preparation Kit for Illumina (Kapa Biosystems, Wilmington, Massachusetts, USA) to build the DNA library. Fragments in the DNA library were PCR amplified and captured by Agilent SureSelect XT2 Target Enrichment System (Agilent Technologies, Inc., Santa Clara, CA, USA). After purification and quantification, DNA was sequenced with 150-bp paired-end reads on the Illumina HiSeq X Ten platform (Illumina, Inc., San Diego, CA, USA), according to the standard manual.

Raw data were filtered and aligned against the human reference genome (hg19) using the BWA Aligner (version: 0.7.15-r1140). GATK software (version: vnightly-2017-03-03-gc6cac2d) was used to call the single-nucleotide polymorphisms. Variants were annotated by ANNOVAR (version: 2017Jun01). Patient 1 was analyzed using the whole-exome sequencing, and the other two patients were analyzed with the panel about seizure-causing genes. Effects of single-nucleotide variants were predicted by in silico algorithms such as Mutation Taster2, MutPredLOF, CADD, NetGene2 Server, NNSplice, and FATHMM-Indel [10,11,12,13,14,15]. All variants were classified according to the American College of Medical Genetics and Genomics (ACMG) standards [16].

Sanger sequencing was also performed to confirm the candidate causal genes discovered by NGS and to carry out the co-segregation analysis. The primers were designed using Primer Premier 6.0 (Premier Biosoft, San Francisco, CA, USA). Amplified target genes by PCR were purified and sequenced by ABI 3730XL DNA Sequencer (Applied Biosystems, Foster City, CA, USA). Sanger sequencing results were analyzed by Chromas Lite v2.01 (Technelysium Pty Ltd., Tewantin, QLD, Australia).

### 2.3. Deposition of Sequences

All three *CHD2* variants have been deposited in the NCBI’s Sequence Read Archive (SRA) database. The reserved accession numbers are SRR18106160, SRR18106159, and SRR18106158, respectively.

### 2.4. Molecular Analysis

RNA was extracted from the peripheral blood sample of patient 1(additional samples are not available for patients 2 and 3 for further experiments) using TRIzol Reagent (ThermoFisher, 10296028). Briefly, RNA was released in the aqueous phase after the blood sample was mixed with TRIzol Reagent and lysed by chloroform. Then RNA was washed with alcohol. After being dissolved in RNase-free water and quantified, RNA was reversed transcribed to cDNA using the GoScript™ Reverse Transcriptase (Promega, A5003). PCR amplification and agarose gel electrophoresis were then performed for the splicing-site variant, using proper primers (F: GCAGAGACCACGATTTGTAGC, R: GGCTCTAGCACCTTATGAAGACTC).

## 3. Results

### 3.1. Clinical Features

Three patients were included in this study, clinical presentations of which were partly heterogeneous (Table 1).

Patient 1 was a twelve-year-two-month-old boy born at full-term via cesarean delivery. At the ages of two years and three months and three years and six months, he suffered similar febrile convulsions. Symptoms included upturned eyes, pale face, cyanosis of lips, clenched teeth and hands, and muscular hypertonia in limbs, which resolved spontaneously within 3–4 min. EEG and brain magnetic resonance imaging (MRI) results were normal. Furthermore, the patient did not receive any treatments during these two seizures. At the age of three years and seven months, the patient presented with frequent convulsions without fever. Symptoms were similar to those described before. The results of brain MRI were normal, while interictal video EEGs revealed numerous multifocal epileptic discharges on both sides during the sleep and awake period. The diagnosis of epilepsy was confirmed, and he was treated with topiramate. The patient remained seizure-free for only about eight months before the febrile convulsions recurred. Nitrazepam was added, while the patient presented with similar symptoms as before for the next two years. When the patient was six years and seven months, he was treated with a combination of valproic acid, topiramate, and lamotrigine, supplemented with a ketogenic diet. The treatment was effective, and the patient has never suffered seizures until now. During the treatment for epilepsy, the patient underwent another two EEG tests, the results of which were abnormal (Table 1). The Wechsler Intelligence Scale for Children-IV was used to assess intellectual disability (ID) of patient 1 at ten years and four months old, which showed moderate (IQ = 40) ID.

Patient 2 was an eight-year-eleven-month-old girl with normal birth history. She had slender fingers and presented with hypotonia in infancy. At the age of two years and eleven months, the patient had her first seizure, which was thought to be caused by fever. At the age of five years, the patient presented with another seizure, which lasted for 2–3 min. Symptoms include an upturned right eye, cyanosis, closed teeth, foaming at the mouth, firm and symmetrical limbs, and urinary incontinence. Brain MRI showed an enlarged subarachnoid space in the frontal and temporal regions. VEEG showed a large number of 2–3 Hz polyspike waves accompanied by short bursts of multiple spike waves. The diagnosis of epilepsy was established, and the patient was treated with valproic acid (15 mg/kg/day). However, during the next month, the patient experienced four seizures. More valproic acid was given (30 mg/kg/day) and no seizures occurred. At the age of five years and three months, the patient developed seizures due to a cold, which was milder than before and was controlled by valproic acid. A test of the Wechsler Intelligence Scale for Children-IV was conducted, which showed mild (IQ = 66) ID.

Patient 3 is an eleven-year-three-month-old boy with a very different presentation from patients 1 and 2. The patient was first admitted to the hospital at the age of six years and four months, due to “two days of fever and eight seizures.” Computed tomography (CT) and MRI of the brain were performed, results of which showed an abnormal density shadow in the right basal ganglia area and right thalamus with CT value 60–68 HU, and a space-occupying lesion of the right thalamus, respectively. The patient was treated with adenosine triphosphate (ATP) intravenously and was stable without fever or seizures. One month later, the patient was readmitted to the hospital. The EEG results showed diffuse polyspike waves and slow spike waves. However, no antiepileptic drugs have been given to treat seizures until the patient was eight years and four months old. The combination of oxcarbazepine and levetiracetam was effective, and the patient did not have any seizures afterward. During these years, three examinations (one MRI and two CT) of the brain were performed, the results of which were all similar to the previous findings. Although no biopsy was performed, the nature of the patient’s brain pathology was unknown. Considering similar findings of two brain MRIs and three brain CTs, calcification was more likely. When the patient was seven years and one month old, he was classified as moderately developmentally delayed, according to the 0~6-year-old pediatric examination table of neuropsychological development (Institute of Pediatrics of Capital Medical University, CNBSR 2016).

### 3.2. Sequencing Results

**Figure 1:**
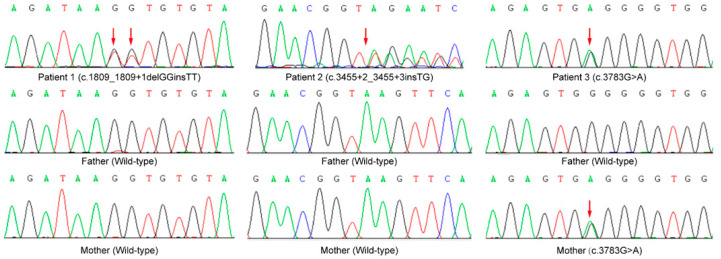
*CHD2* variants validated by Sanger sequencing.

Next-generation sequencing was performed to identify the likely pathogenic variants associated with EEs. Sanger sequencing was then performed to validate the candidate causal variants and to analyze the co-segregation of the causal variants in the patients’ families. Finally, three likely pathogenic variants of *CHD2* (NM_001271.3) were detected, which were predicted to cause their epileptic syndromes. Two variants have never been reported, and the other variant was only reported with the genotypes in our previous study (Figure 1) [17].

Patient 1 carried a *de novo* deletion–insertion (delins) variant c.1809_1809+1delGGinsTT (p.?) (PS2), which was predicted to affect the splicing and maturation of mRNA at the splicing donor site of intron 15, resulting in the loss of protein function (PVS1). Patient 2 had a *de novo* variant c.3455+2_3455+3insTG (PS2), which was also predicted to result in an improper splice in the splicing donor site of intron 27. Meanwhile, a maternal variant c.3783G>A (p.W1261*) was identified in patient 3 and was predicted to be pathogenic by Mutation Taster2, MutPredLOF and CADD (Table S1). This nonsense variant introduces a premature terminator and results in a truncated protein, leading to the loss of protein function (PVS1). All these variants were absent from healthy controls from ExAC and gnomAD (PM2). Therefore, variants c.1809_1809+1delGGinsTT (p.?), c.3455+2_3455+3insTG and c.3783G>A (p.W1261*) were classified as pathogenic (PVS1+PS2+PM2), likely pathogenic (PS2+PM2+PP3) and likely pathogenic (PVS1+PM2), respectively (Table S1).

### 3.3. Molecular Experiments

**Figure 2:**
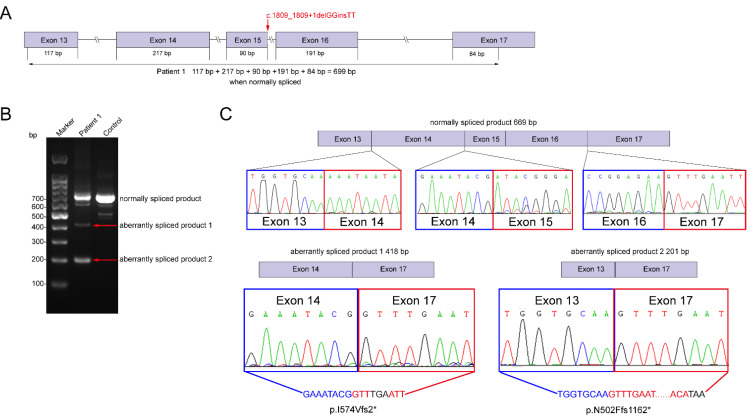
Molecular analysis of patient 1’s *CHD2* variants. (**A**) Schematics showing the location of primers and the variant c.1809_1809+1delGGinsTT within the *CHD2* gene. (**B**) Agarose gel electrophoresis of amplified complementary DNA from patient 1 and the normal control, with the position of normally spliced (669 bp for line 3) and aberrantly spliced products (lines 2) indicated. (**C**) Sanger sequencing of the normally and aberrantly spliced products from Patient 1. The variant affects the normal splicing of mRNA, resulting in the skipping of exons 15–16 and 14–16.

To investigate whether the splicing-site-related variant would affect the splicing sites of *CHD2*, RT-PCR amplification using specific primers flanking exons 13 to 17 on cDNA was performed in patient 1 (Figure 2A). Agarose gel electrophoresis of amplified complementary DNA from patient 1 (Figure 2B, line 2) as well as normal control (Figure 2B, line 3) showed normally spliced (699 bp) and many different aberrantly spliced products. Sanger sequencing results showed that patient 1 had normally spliced mRNA and additional RNA species (aberrantly spliced products 1 and 2), which lacked exons 15–16 and 14–16, respectively (Figure 2C). The delins variant c.1809_1809+1delGGinsTT (p.?) introduced premature stop codons (p.I574Vfs2* and p.N502Ffs1162*), resulting in truncated proteins with 573 and 502 correct amino acids. Truncated proteins with an unknown functional tail resulted in the haploinsufficiency of CHD2, which could cause disease in the patient.

## 4. Discussion

EEs are a group of epilepsy disorders characterized by seizures and cognitive arrest or regression, which often carry a poor prognosis [18]. With the advances in NGS technology, EEs are reported to be caused by pathogenic variants in several genes [3]. These genetic discoveries revolutionize the clinical practice, providing a molecular basis for the diagnosis of EEs [19]. The researches on the pathogenicity of epilepsy-related variants are critical for counseling families about the recurrent risk and developing the molecular-specific treatment.

*CHD2* encodes the chromodomain-helicase-DNA-binding protein 2, which comprises 1828 amino acids. It belongs to the chromodomain helicase DNA-binding family, regulating the gene expression by remodeling the chromatin structure and influencing histone acetylation [5]. Correlation with epilepsy (DEE94) was first reported in 2009 [6]. To date, over 80 disease-causing variants have been reported, most of which were assumed or confirmed *de novo*. Only four variants have been reported to be inherited from the parents. One missense variant (c.653C>T, p.P218L) and one splicing-site variant (c.5153+2T>C) were inherited from the unaffected mother and father [20,21]. While one nonsense variant (c.628G>T, p.E210*) was identified in an affected mother and her daughter [5]. The same missense variant (c.854C>T, p.A285V) was identified in a brother and sister with DEE94, who inherited the variant from their mother. However, the clinical phenotypes were unknown [22]. In our study, patient 3 inherited the nonsense variant (c.3783G>A, p.W1261*) from his clinically unaffected mother. Although we were unable to obtain additional samples from this patient and his mother to experimentally demonstrate the pathogenicity of this variant. Considering the patient’s seizures and developmental delay, and the fact that there are no other variants that could cause these phenotypes in the patient, we considered that the c.3783G>A variant of *CHD2* was a likely pathogenic variant and was responsible for the patient’s phenotypes. More cases with detailed clinical information are needed to determine if the incomplete penetrance or phenotypic heterogeneity of *CHD2* variants (the patient’s parental phenotype was too mild to be detected) exist, or if there are other unknown causative genes that can cause seizures and developmental delays.

Among the different variant types, the splicing variant was more common in our cohort (2/3). Frameshift, one of the most common variant types reported in previously published individuals, however, was not detected in our patients. It has been predicted that the haploinsufficiency of CHD2 could cause epilepsies in children [1]. A similar result was detected in patient 1, who carried a delins variant c.1809_1809+1delGGinsTT (p.?). Agarose gel electrophoresis of amplified complementary DNA showed additional RNA species associated with the normally spliced RNA. Sanger sequencing was performed, and we found out-of-frame deletions of exons 15–16 and 14–16 in different aberrantly spliced products. Premature stop codons were introduced, resulting in truncated proteins with 573 (p.I574Vfs2*) and 502 (p.N502Ffs1162*) correct amino acids, respectively. The aberrant proteins might be degraded by the proteasome, or the mutated proteins may not be fully functional, both of which would cause the haploinsufficiency of CHD2 and lead to neurodevelopmental disorders as a result, as speculated by Suls et al. [23]. Furthermore, it seemed that the ratio of wild-type to other mutated mRNA was 1:1, which means that these two mRNA types are exclusively derived from the normal c.1809_1809+1GG allele and the mutant c.1809_1809+1TT allele, respectively. Thus, the variant c.1809_1809+1delGGinsTT (p.?) abolishes normal splicing of exons 13 to 17, introducing premature termination of translation, thereby confirming the pathogenicity of this variant.

**Figure 3:**
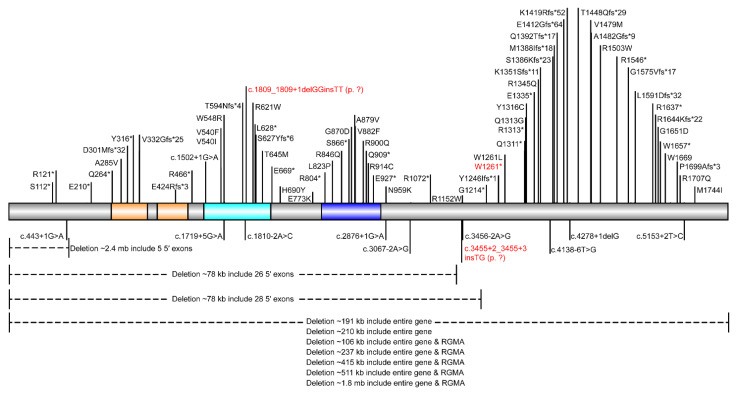
Schema of CHD2 with disease-causing mutations reported in patients. CHROMO domains, helicase ATP-binding domain, and helicase C-terminal domain were colored in orange, cyan, and blue, respectively. Novel pathogenic/likely pathogenic variants reported in this study were presented in red texts.

The disease-causing mutations of *CHD2* reported in the HGMD were collected (Figure 3). However, the different variant types in *CHD2* were not clustered in any definite pattern within the gene. The reported missense variants do not cluster in functional domains of the protein either (Figure 3). Thus, the correlation between genotype and phenotype remained unknown. More cases and functional studies are needed to clarify the exact mechanism of DEE94 caused by *CHD2* pathogenic variants.

In our study, patients 1 and 2 presented with their first seizures at the age of about two years, while the age at seizures onset of patient 3 was six years. It has been reported that the first seizure presented in *CHD2* patients was usually before they reached two years of age [24]. It seemed that DEE94, caused by a heterozygous pathogenic variant in the *CHD2* gene, might also occur in the later years of childhood. Fever was a susceptibility factor in seizure induction. The seizure onset of our three patients and the other fourteen patients (a total of seventeen) were induced by fever [21,23,24,25,26]. Most patients with *CHD2* variants presented multiple seizure types, especially generalized tonic-clonic seizure (GTCS) (32/59) and myoclonic seizure (MS) (28/59) (Table 1). It is interesting that in our study, only patient 3 patients showed GTCS and MS. Patient 2 had MS. While patient 3 only presented FS. Phenotypes caused by different *CHD2* variants were likely to be heterogeneous. The light could be another factor in inducing seizures. Fifteen of eighty-nine patients reported previously were photosensitive [21,24,27], and a partial (50%) knockdown of functional CHD2 proteins markedly enhanced Zebrafish larval photosensitivity [20]. However, in our study, patient 1 underwent intermittent photic stimulation (IPS) and showed no photoparoxysmal response (PPR). Patient 2 had an enlarged subarachnoid space in the frontal and temporal region, which was not an epileptogenic lesion. In previous literature, some patients with *CHD2* variants also had abnormal brain MRI findings: one patient had cerebellar vermis hypoplasia, and three patients presented with progressive atrophy, whose ages were 10 years or older at the last follow-up [6,24]. A right thalamic occupancy was found in patient 3, which was presumed to be calcification of unknown origin. The lesion was discovered during the patient’s first hospital visit and there were no definite changes in multiple brain MRI and CT examinations. Therefore, the lesion was determined to be non-acute and non-progressive. The relationship of this lesion to patient 3’s clinical presentation is difficult to infer. Perhaps the lesion is unrelated to the existing disease (more likely), perhaps it is a structural factor contributing to the patient’s seizures, or perhaps it is a rare phenotype caused by the patient’s *CHD2* variant. Regular examination of brain MRI is necessary and more cases are needed for the further exploration of *CHD2* variants and the related disease.

All three patients reported in our study were followed-up and were all seizure-free for over one year. When they became seizure-free, their ages ranged from five years and one month to eight years and four months. However, it seems difficult to control seizures caused by *CHD2* variants, as seizure freedom could only be achieved temporarily in our patients. Seizure reoccurrences are common and usually require multiple medications to control them. Valproic acid (patient 2), oxcarbazepine, and levetiracetam (patient 3) could be chosen to cure epilepsy, which had been effectively used in our patients. Valproic acid and lamotrigine were the most used drugs, which have been given to 34 (92%) and 21 (57%) of 37 patients who were described previously with a detailed drug history (Table 1). Lamotrigine, levetiracetam, and valproic acid have been used alone to treat patients’ seizures (lamotrigine for one patient, levetiracetam for one patient, and valproic acid for five patients including our patient 2). Seizures were well-controlled in patients treated with lamotrigine and levetiracetam. While for patients treated with valproic acid, seizures were well-controlled in three patients, partially controlled in one patient, and uncontrolled in one patient. The combination of valproic acid and levetiracetam seems to be more effective, as seizures of four patients have been well-controlled. However, no particularly clear evidence has shown which antiepileptic drug or drugs are most effective. There may be no known single or combination of antiepileptic drugs that could control refractory seizures in patients who carried *CHD2* variants. More cases with detailed treatment histories were needed for the treatment and prognosis of *CHD2*-related epilepsy.

## 5. Conclusions

Here, we describe three patients with novel likely pathogenic *CHD2* variants. The spectrum of *CHD2* pathogenic variants is extended and we provide additional evidence that variants associated with splicing sites can affect the normal splicing of *CHD2*, causing disease in patients (patient 1). Brain atrophy (patient 2) and incomplete penetrance (patient 3) could exist, and there might be a wider phenotype in epilepsy caused by *CHD2* variants. We also found that seizure reoccurrences are common and could not be well-controlled easily. Description of additional patients with *CHD2* variants is needed to further explore these assumptions.

## Figures and Tables

**Table 1:** Clinical and genetic characteristics of patients with *CHD2* variants.

Proband	1	2	3	Patients Reported Previously (*n* = 65)
Gender	M	F	M	M (38)/F (26)/NK (1)
Variants	c.1809_1809+1delGGinsTT (p. ?) 15:93498742:93498743:GG:TT	c.3455+2_3455+3insTG (p. ?) 15:93534749:93534749:T:TTG	c.3783G>A (p.W1261*) 15:93540531:93540531:G:A	Nonsense (17)/Frameshift (18)/Splice (6)/Missense (15)/Deletion (9)
Inheritance	*De novo*	*De novo*	Maternal	*De novo* (51)/Paternal (3)/Maternal (1)/NK (10)
Family History of Epilepsy	-	+	-	Yes (8)/No (23)/NK (34)
ACMG classification	P (PVS1+PS2+PM2)	LP (PS2+PM2+PP3)	LP (PVS1+PM2)	P (42)/LP (21)/VUS (2)
Onset ages	2y3m	2y11m	6y4m	6m-10y5m
Brain Imaging (MRI)	-	Enlarged subarachnoid space in the frontal and temporal region	Space-occupying lesion of the right thalamus	Normal (25)/Abnormal (10)/NK (30)
				
*Seizure Types (patients can have multiple seizure types)*				Patients reported with different seizure types (*n* = 59)
-Seizures	+	+		Solo (1)/Combined (8)
-Epileptic spasm	+			Solo (0)/Combined (2)
-Status epilepticus				Solo (0)/Combined (5)
-Atonic seizures				Solo (0)/Combined (11)
-Clonic seizures				Solo (0)/Combined (2)
-Tonic seizures				Solo (1)/Combined (7)
-Generalized tonic seizures				Solo (0)/Combined (2)
-Myoclonic seizures	+			Solo (2)/Combined (28)
-Focal myoclonic seizures				Solo (0)/Combined (1)
-Generalized myoclonic seizures				Solo (1)/Combined (1)
-Focal seizures	+			Solo (1)/Combined (5)
-Focal impaired awareness seizures				Solo (0)/Combined (1)
-Absence seizures				Solo (4)/Combined (11)
-Absence seizures (Atypical)				Solo (0)/Combined (10)
-Absence seizures (with Eyelid myoclonia)				Solo (0)/Combined (5)
-Generalized seizures	+			Solo (1)/Combined (2)
-Generalized myoclonic-absence seizures				Solo (0)/Combined (3)
-Generalized myoclonic-atonic seizures				Solo (0)/Combined (5)
-Generalized myoclonic-atonic-absence seizures				Solo (0)/Combined (3)
-Generalized myoclonic-clonic seizures				Solo (0)/Combined (1)
-Generalized tonic-clonic seizures	+	+		Solo (6)/Combined (32)
-Febrile seizures	+	+	+	Solo (0)/Combined (8)
Fever Sensitivity of Seizure Onset	+	+	+	Yes (14)/No (14)/NK (31)
Developmental Delay Before Epilepsy	+	-	+	Yes (17)/No (8)/NK (34)
EEG/VEEG	Multifocal epileptic discharges at 3y7m; High-amplitude sharp and slow wave, slow spike wave, slow polyspike wave at 5y3m; Generalized spike waves, slow spike wave, slow polyspike wave at 7y3m	Focal epileptic discharges, 2–3Hz polyspike wave at 5y	Diffuse polyspike wave, slow spike wave at 6y5m	Abnormal(33)/Normal(0)/NK(26)
Photosensitivity	-	NK	NK	Yes (16)/No (2)/NK (41)
				
*Treatment*				Patients reported with detailed medication history (*n* = 37)
-Bromide				Solo (0)/Combined (2)
-Clobazam				Solo (0)/Combined (7)
-Clonazepam	+			Solo (0)/Combined (10)
-Ethosuximide				Solo (0)/Combined (4)
-Lamotrigine	+			Solo (1)/Combined (14)
-Levetiracetam			+	Solo (1)/Combined (20)
-Oxcarbazepine			+	Solo (0)/Combined (4)
-Phenobarbital				Solo (0)/Combined (3)
-Phenytoin				Solo (0)/Combined (1)
-Rufinamide				Solo (0)/Combined (2)
-Topiramate	+			Solo (0)/Combined (10)
-Valproic acid	+	+		Solo (4)/Combined (34)
-Vigabatrin				Solo (0)/Combined (1)
-Zonisamide				Solo (0)/Combined (4)
-Ketogenic diet	+			Solo (0)/Combined (5)
Seizure Remission (age)	Yes (6y7m)	Yes (5y1m)	Yes (8y4m)	Yes (18)/Partially (2)/No (12)/NK (5) Effective medication or combination: Valproic acid + Levetiracetam (4); Valproic acid + Lamotrigine (2); Valproic acid (2); Levetiracetam (1); Lamotrigine (1); Valproic acid + Clonazepam (1); Valproic acid + Ethosuximide + Lamotrigine (1); Valproic acid + Clobazam + Lamotrigine (1); Valproic acid + Clobazam + Rufinamide (1); Valproic acid + Levetiracetam + Topiramate (1); Valproic acid + Levetiracetam + Topiramate + Clobazam (1); Valproic acid + Levetiracetam + Topiramate + Lamotrigine + Zonisamide (1); Valproic acid + Clonazepam + Lamotrigine + Levetiracetam + Oxcarbazepine + Phenobarbital + Topiramate (1)
				
*Development (Psychomotor and Cognition)*				Patients reported previously (*n* = 65)
-ID	+	+		*n* = 27
-Delayed psychomotor development	Delayed language and motor development		Moderate developmental retardation	*n* = 46
-Psychomotor regression		Intellectual regression		*n* = 17
*Behavior*				
-ASD				*n* = 23
-ADHD				*n* = 7
-Aggressive behavior				*n* = 17
-Psychotic features				*n* = 9
-Bruxism				*n* = 2
-Stereotypic movements				*n* = 4
-Limited social skills				*n* = 3
-Short attention span				*n* = 2
Dysmorphic features	Microcephaly	Slender fingers		Normal (52)/Abnormal (13)
Other		Hypotonia in infancy		Hypotonia (7), scoliosis (5), feeding difficulties (2), visual disability (3), short stature (4), gait (7), ataxia (7)

**Key:** M = male, F = female, m = month, y = year, P = pathogenic, LP = likely pathogenic, NK = Not known, EEG = electroencephalography, VEEG = video electroencephalography, MRI = magnetic resonance imaging, PPR = photoparoxysmal response, ID = intellectual disability, ASD = autism spectrum disorder, ADHD = attention deficit hyperactivity disorder.

## Data Availability

The data presented in this study are available on request from the corresponding author. The data are not publicly available due to the request of the participants.

## Supplementary Materials

The following supporting information can be downloaded at: https://www.mdpi.com/article/10.3390/genes13050908/s1, Table S1: Bioinformatic analysis of different *CHD2* variants; Table S2: Clinical features of patients reported in previous literature with *CHD2* disease-causing mutations (DM) (according to HGMD).
