# Supplementary material for: Novel Loss-of-Function Variants in CHD2 Cause Childhood-Onset Epileptic Encephalopathy in Chinese Patients
Source: Genes (Basel). 2022 May 19;13(5):908. doi: 10.3390/genes13050908 (PMC9140428; doi:10.3390/genes13050908)
Supplement: Supplementary file 1 [file genes-13-00908-s001.zip › Supporting information Table S1.pdf]

Table S1. Bioinformatic analysis of different *CHD2* variants.

Patient	Mutation	Exon /Intron	Inheritance	Mutation Taster2	MutPredLOF	CADD	NetGene2 Server	NNSplice	FATHMM-Indel	Classified according to ACMG guidelines
1	c.1809_1809+1delGGinsTT (p. ?)	Exon 15 & Intron 15	*De novo*	N/A	N/A	Pathogenic (32)	Influenced	Influenced	N/A	Pathogenic (PVS1+PS2+PM2)
2	c.3455+2_3455+3insTG Splice	Intron 27	*De novo*	N/A	N/A	Pathogenic (25.1)	Influenced	Influenced	Pathogenic (0.986)	Likely pathogenic (PS2+PM2+PP3)
3	c.3783G>A p.W1261X	Exon 30	Maternal	Disease causing (1.00)	Possibly damaging (0.50<0.62<0.70)	Pathogenic (40)	N/A	N/A	N/A	Likely pathogenic (PVS1+PM2)
